# A Simple, Inexpensive Method for Mark-Recapture of Ixodid Ticks

**DOI:** 10.1093/jisesa/ieaa052

**Published:** 2020-11-02

**Authors:** Alexis White, Robin Minch, Lindsey Bidder, Holly Gaff

**Affiliations:** 1 Department of Biological Sciences, Old Dominion University, Norfolk, VA; 2 Quantitative Disease Ecology and Conservation (QDEC) Lab Group, Department of Geography, University of Florida, Gainesville, FL; 3 Emerging Pathogens Institute, University of Florida, Gainesville, FL; 4 Department of Biology, College of Charleston, Charleston, SC; 5 School of Mathematics, Statistics and Computer Science, University of KwaZulu-Natal, Durban, South Africa

**Keywords:** mark-recapture, capture-mark-recapture, Ixodid, ticks

## Abstract

Mark-recapture techniques have been widely used and specialized to study organisms throughout the field of biology. To mark-recapture ticks (Ixodida), we have created a simple method to mark ticks using nail polish applied with an insect pin secured in a pencil that allows for a variety of questions to be answered. For measuring tick control efficacy, estimating population estimates, or measuring movement of ticks, this inexpensive mark-recapture method has been easily applied in the field and in the lab to provide useful data to answer a variety of questions about ticks.

Mark-recapture is a technique that has been used by biologists in nearly every field from botany to wildlife (Pollock 1980, Alexander et al. 1997, Schwarz and Seber 1999). It can be used for several metrics or indicators including population estimates, survival, and dispersal. Depending on the study organism and the information needed, different methodologies have been developed.

Ticks are vectors of disease-causing pathogens and monitoring them is important to protecting humans, domesticated animals, and wildlife. As early as the 1930s, biologists were marking ticks to better understand their ecology (Philip 1937). Since then mark-recapture of ticks has been used to study movement of ticks (Sonenshine and Levy 1971, 1972; Sonenshine 1972, 1973; Wilson et al. 1972; Conlon and Rockett 1982; Gray 1985; Lane et al. 1985, 2009; Solberg et al. 1992; Goddard 1993; Goddard et al. 2011), estimate populations or analyze population dynamics (Smith et al. 1946, Sonenshine 1972, Garvie et al. 1978, Conlon and Rockett 1982, Eads and Smith 1983, Koch 1987, Daniels et al. 1989, Carroll et al. 1991, Goddard and Goddard 2008), and measure survival (Koch 1987, Daniels et al. 1989, Gaff et al. 2015, Bidder 2016, White 2019). The method used to mark the ticks has varied from paints (Philip 1937, Smith et al. 1946, Gray 1985, Koch 1987, Carroll et al. 1991), radioisotope-tagged markers (Sonenshine and Levy 1971, 1972; Sonenshine 1972, 1973; Garvie et al. 1978), fluorescent powder (Wilson et al. 1972, Conlon and Rockett 1982, Daniels et al. 1989, Falco and Fish 1991, Solberg et al. 1992, Goddard 1993, Kramer et al. 1993, Lane et al. 2009, Goddard et al. 2011), liquid paper (Lane et al. 1985), and nail lacquer, the technique described in detail here (Gaff et al. 2015, Bidder 2016, White 2019).

Insects have been studied considerably with mark-recapture. Various marking methods have been developed and adapted over time to better ask questions about insects ranging from population dynamics to trophic-level interactions (Hagler and Jackson 2001). Hagler and Jackson 2001 describe the ideal mark to not inhibit the study organism should be environmentally safe, cost-effective, and easy to apply. Our method of marking ticks does just that.

The protocol presented here describes a mark-recapture method for ticks that could be applied to other invertebrates, such as insects. It is a low-cost method that can provide detailed capture histories for both nymphal and adult ticks as well as mark unique individuals. This method of mark-recapture is also safe for the environment, does not harm the tick, and is easy to teach to interns. Target users for this protocol would include tick biologists but also other invertebrate biologists where this method of marking could be useful.

## Experimental Design

In every study, the very important initial step is collecting ticks. Ticks can be sourced from lab colonies or collected from the field through flagging or dragging. Materials necessary for this marking technique include nail polish, a pencil with a rubber eraser, insect pins size 000, blue painter’s tape, and a hard surface. Inexpensive nail polish has been shown to last in the field for at least 10 wk (Bidder 2016). The exact quantity of nail polish required for the experiment will vary depending on the study. The number of colors required also varies with the specific project, e.g., marking ticks each capture event with multiple events in a single day will require more color variety than only marking ticks once a week. A pencil with a rubber eraser is needed to design a ‘paint brush’ tool with the insect pins. A single pin is pushed into the eraser of the pencil to act like the brush and add comfort to the painter’s ability to mark the tick. Blue painter’s tape is desired over scotch or masking tape as the adhesiveness will hold the ticks in place but also allow removal without damaging the tick itself. Lastly, we found that a storage clip board was very useful for storing all of the paint colors as well as the tools necessary for painting the ticks. Estimated costs for all of the equipment are available in Table 1.

**Table 1.** List of materials needed for this mark-recapture technique and estimated cost

Material	Quantity	Cost
Nail polish	1	$1
Pencil	10	$1
Insect pins size 000	100	$9
Blue painter’s tape	1	$3
Pointed forceps	1	$2
Storage clip board	1	$10
Total		$26

## Procedure

### Lab Collection

1. Remove ticks from vial individually and place on blue painter’s tape with the dorsal side of the tick exposed.
2. Continue with steps 2–8 below as relevant for the lab study.

### Field Collection

**Fig. 1.**
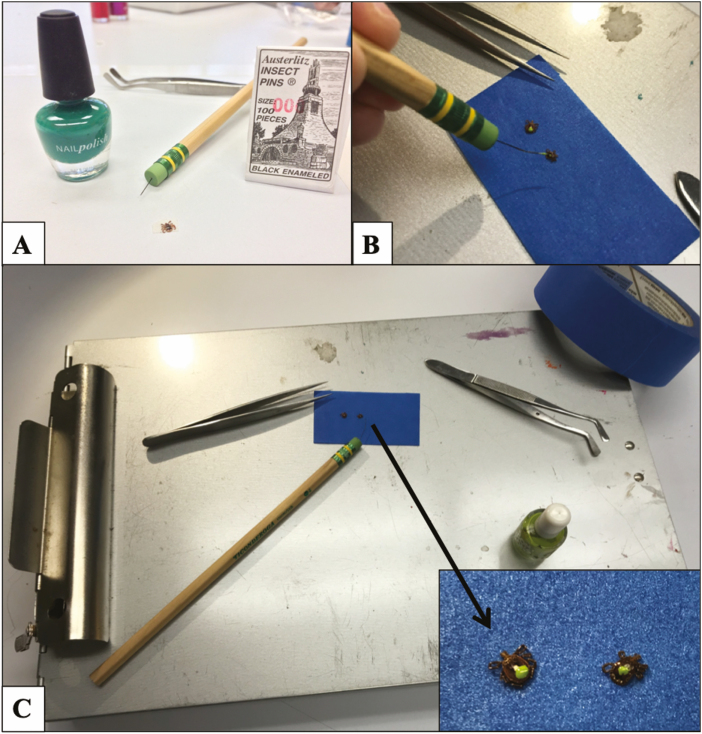
Setup and process of painting ticks with modified paint brush of pencil and insect pin. (A) A size 000 insect pin is secured in the eraser of pencil. (B) This modified paint brush is used to paint the tick. (C) A storage clipboard is a convenient tool for mobile applications of this technique. Blue painter’s tape provides an ideal working surface to secure the ticks for painting (shown in inset).

**Fig. 2.**
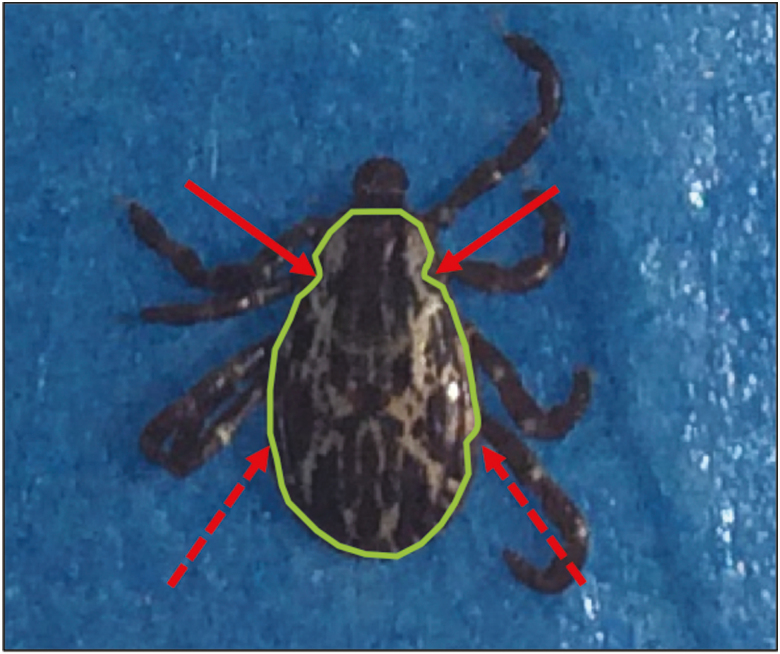
Dorsal *D. variabilis* male with a green polygon indicating where to paint and red arrows indicating areas to avoid. Dotted red arrows point to the approximate location of spiracular plates located ventrally on the tick, and solid red arrows point to the approximate location of the eyes. Both of these organs should remain unpainted so as not to impede the tick’s survival or host-seeking cues.

1. Flag or drag for ticks, stopping every 3 m to remove all ticks from the cloth using fine-pointed tweezers and stick ticks ventrally to a 5-cm piece of blue painter’s tape leaving the scutum free to painted.
2. Once ticks are secured on tape, roll the edges of the tape and stick it to the clipboard (or another hard surface) (Fig. 1). It is important to monitor the ticks at all times on the tape as occasionally a tick will free themselves and begin to crawl away.
3. Before painting the ticks, record all data about the specimens (date, time, transect, species, life stage, fate (whether the tick was a new capture or recapture), what color of paint was currently on the tick if any, and the paint color that will be used to mark the tick).
4. Once data are recorded, use another piece of 5-cm piece of tape and create a paint pallet by sticking the tape sticky side down on the clipboard.
5. Next, use the nail polish brush in the paint bottle to place a drop of the desired paint color on the tape paint pallet and close the nail polish bottle.
6. Use the modified paint brush (created by a pencil and insect pin) to get a small dot of paint on the end of the pin (Fig. 1), and place the small dot of paint on the scutum of the tick. Use caution when painting all ticks to avoid spiracular plates and the distal ends of the legs (Fig. 2).
7. Wait a few seconds for the nail polish to dry, then the tick may be removed with pointed forceps and placed in the desired location. In our field-based studies ticks were returned to ground level foliage in the approximate location they were collected.
8. Return to step 1 and sample another 3 m to begin the process again.

**Fig. 3.**
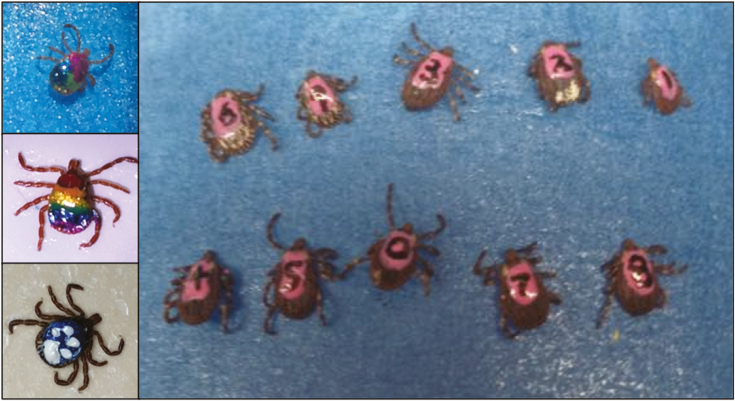
Examples of painted ticks. Ticks can be painted with multiple colors to allow for notation of multiple recapture events. Ticks can also be marked as individuals using a fine-tipped marker over a light paint color.

If individual identification is required, numbers or symbols can be added with fine-tipped permanent marker, or the location of the dot on adult ticks may be used (Fig. 3). On adult tick species with larger scutums (e.g., *Dermacentor variabilis* Say [Ixodida: Ixodidae] or *Amblyomma americanum *Linnaeus [Ixodida: Ixodidae]) numbers 0–99 could be written identifying 100 individuals. Alternatively, an elaborate paint pattern scheme could be used to identify several individuals with paint order on the tick scutum mattering to identify each individual. For example, at maximum on the scutum of an adult *A. americanum* or *D. variabilis* we have painted six different identifiable colors, with only using six different paint colors we would potentially be able to mark 46,656 individuals. Nail polish is readily available in an abundance of colors, and thus several capture events, or even more individual patterns, could be documented on ticks’ scutums (Fig. 3).

Major limitations of this method include color blindness of the observer especially between small dots, and dexterity or steadiness of one’s hand to paint a small surface. Observers must also be able to field identify collected ticks for accurate measurements before marking and releasing. Variation in tick size based on species and life stage can also present limitation of this technique, painting six field identifiable colors on a nymph is not possible. In extremely humid field conditions, the nail polish does not dry as quickly and so the process takes longer. If the tick is accidentally collected dorsally on the tape, the nail polish may fall off. It is useful to have all colors of paint accessible so ticks can be remarked.

## Results

The specific results of this mark-recapture technique depend on the motivation behind a given study. In our lab, we first used mark-recapture to measure the efficacy of a control method, a tick-killing robot (Gaff et al. 2015). Ticks were collected and painted before the TickBot was run, and then after the treatment period, the area was sampled again. If painted ticks were collected from treated areas, then we could measure the TickBot’s effectiveness through a percent reduction compared with recaptures in an untreated area. This method was similarly applied to measure the effectiveness of helmeted guinea fowl (*Numida meleagris* (Linnaeus; Galliformes: Numididae)) as a biological control of ticks. Painted ticks were placed in enclosures with birds and without birds to compare recapture rates (White 2019).

We also used this technique to measure tick movement toward a carbon dioxide source, ticks were painted three different colors based on how close they were initially placed to the carbon dioxide. In addition to the standard painting process, these ticks received individual numbers with a fine-point permanent marker to clearly identify individuals (White 2019).

Using this technique in a field setting, a variety of analyses can be performed beyond control and movement of ticks. Using this mark-recapture technique along a given transect, we were able to calculate the longevity of ticks to quantify how many weeks a tick would remain questing (Bidder 2016, White 2019), as well as the percentage of ticks recaptured and how this varies by site, species, and life stage (Bidder 2016; Supp File 1 [online only]). Furthermore, more elaborate calculations can be used to quantify the probability of survival with Cormack–Jolly–Seber, or population density estimates using a Minimum Number Alive assessment (Supp File 1 [online only]).

## Discussion

Existing mark-recapture techniques have been effective in gathering different information about ticks, but the method presented here provides a simple cost-effective method that can be used by anyone. Previous methods for mark-recapture of ticks, such as radioisotope-tagging or lead-based paints, are now illegal for use in most wildlife settings. Batch marking of individuals using fluorescent powder may still be beneficial for certain questions such as studies requiring individuals to self-mark or where UV light would be beneficial in finding the study organism. However, fluorescent powder does not leave ticks marked for as long as nail polish and has the possibility of marking, unmarked ticks in the substrate if they encounter the residual powder from a marked tick. Our technique described in this protocol will allow for more specificity. Life stage-specific and collection day-specific information can be gathered from this technique. A large variety of nail polish colors are cheap and readily available, and a modification of the pattern upon the tick’s scutum with different colors of paint would easily allow tracking of individuals over time. Although the concept of mark-recapture in ticks is not novel, this method provides researchers a simple and inexpensive method to better understand tick ecology.

## Supplementary Material

ieaa052_suppl_Supplementary_File_1
