# Supplementary material for: A Simple, Inexpensive Method for Mark-Recapture of Ixodid Ticks
Source: J Insect Sci. 2020 Nov 2;20(6):9. doi: 10.1093/jisesa/ieaa052 (PMC7604875; doi:10.1093/jisesa/ieaa052)
Supplement: ieaa052_suppl_Supplementary_File_1 [file ieaa052_suppl_supplementary_file_1.docx]

**Mark Recapture Analysis Case Study: Percent Recapture, Longevity, Cormack-Jolly-Seber, and Minimum Number Alive**

The mark-recapture method outlined in the paper was used to compare the longevity, survival, and population densities of multiple ixodid tick species across six field sites in southeastern Virginia from 2013-2018. It was hypothesized that ecosystems regularly disturbed by humans would have a larger tick population than ecosystems with minimal human contact. By increasing the area of ecotones through habitat fragmentation, humans may inadvertently produce better habitats for ticks. Ecotones have been associated with an increased abundance of ticks (Boyard et al. 2007), and logging activity increasing the number of ecotones in the Tula region of Russia may have contributed to the observed increase in tick abundance from 1991-2011 (Korotkov et al. 2015).

**Methods**

***Field Collection***

These data were collected from 2013-2018 (with the exception of 2017) at six different sites used in different years detailed in Figure S1. All ticks were collected through standard flagging methods along designated transects at each of the field sites. The flagger would stop every 3m, record the necessary data: date, time, transect, species, life stage, fate (whether the tick was a new capture or recapture), what color of paint was currently on the tick if any, and the paint color that will be used to mark the tick. The ticks were then painted, the flagger would wait a few seconds for the paint to dry then ticks were placed back at their approximate sampling locations on top of ground level foliage such as a fern. The flagger then proceeded to sample along the rest of the transect repeating the process every 3m. Different colors of paint were used for each day of sampling so that capture histories could be created for the given ticks (1 for captured, 0 for not captured). The field sites were not consistently sampled between years and thus this is to remain a case study of how mark-recapture can be analyzed. Ideally, we would have consistent data collection between sites and years.

***Field Site Classification***

To explore the effect of human disturbance on tick populations, the six field sites in southeastern Virginia were each classified as disturbed or undisturbed. A wildlife preserve in the City of Portsmouth (PT2) is an area open to the public with multiple trails, while the two sites in the City of Chesapeake (CH1, CH2) are owned by The Nature Conservancy and are not open to the public other than a local hunt club. The site in the City of Newport News (NN1) is a large municipal park and the studied transect was along a service road for the park. The site in the City of Hampton (HM1) is a transect within a closed canopy forest on government property. The second site in the City of Portsmouth (PT3) is a private residence less than 400m from PT2, and the transect was located in the backyard of this property. The six sites were classified into disturbed sites (PT2, NN1, PT3) and undisturbed sites (CH1, CH2, HM1), based upon whether or not humans actively maintain the ecotone along the studied transect. Data from PT2 and CH1 in 2018 were also used to compare the probability of recapture and survival for ticks at both sites.

***Analysis Techniques***

Four metrics were used to analyze these mark-recapture data. Percent recapture was used as an initial analysis to look at how many ticks of each species were recaptured. Second, the data were analyzed to see how long ticks remain at the field site between initial capture and final recapture, which we describe here as longevity. Then, Cormack-Jolly-Seber was used to estimate the probability of recapture and survival for the ticks. Lastly, we used minimum number alive to estimate the density of ticks. Below we go into greater detail about how each of these analysis techniques were used and our results.

**Analysis Techniques and Results**

***Percent Recapture and Longevity***

Across all sites and years, a total of 1,118 ticks were captured from five species: lone star ticks (*Amblyomma americanum* (Linnaeus; Ixodida: Ixodidae)), Gulf Coast ticks (*Amblyomma maculatum* (Koch; Ixodida: Ixodidae)), American dog ticks (*Dermacentor variabilis* (Say; Ixodida: Ixodidae)), *Ixodes affinis* (Neumann; Ixodida: Ixodidae), and blacklegged tick*s* (*Ixodes scapularis* (Say; Ixodida: Ixodidae))*.* Of these individuals, 100 were recaptured for an 8.2% recapture rate overall. Female *D. variabilis* had the highest recapture rate with 11 of the 22 marked individuals recaptured. Male *A. americanum* had the second highest recapture rate with 39 of 290 marked individuals later being recaptured (Figure S2). Of those recaptured, the time between the initial capture and the final recapture varied from hours to ten weeks (Figure S3).

***Cormack-Jolly-Seber***

To estimate tick population density, data from PT2 and CH1 in 2018 were fitted to Cormack-Jolly-Seber (CJS) models using R (R Core Team 2019), RMark (Laake 2013), and MARK (White and Burnham 1999). These CJS models were then used to determine the probability of recapture and survival for individuals, and then we compare these probabilities between both sites. Larger populations were expected to have lower recapture rates and higher survival probabilities. The CJS model allows for estimations of open populations, and uses all weeks studied to estimate recapture and survival probabilities. Individuals were given a binary capture history and described by species and life stage (adult female, adult male, or nymph). Models were constructed with a constant probability of capture life stage, species, and the date as possible explanatory variables. Each variable, as well as each combination of variables interacting, was used to explain either the capture or survival probability for a total of 67 models.

The CJS models that best fit the 2018 data (Table S1) were determined with Akaike’s Information Criterion with correction (AICc) values to determine which parameters best explained the data. AICc values were used due to small sample size (Hurvich and Tsai 1989). The probability of survival at both sites was estimated with a weighted average for the best fitting models. The averaged CJS models were then used to determine the recapture and survival probabilities of individuals at the two sites. Afterwards, a Chi-square test was used to compare probabilities between the two sites.

For PT2 in 2018, the survival probability was mostly constant, while survival was influenced by all variables tested at CH1 (Figure S4). Amongst species shared between the two sites, there was a significant difference in survival (χ^2^=748, df=20, p=0.005), with ticks at PT2 having a higher probability of survival.

Overall, tick recapture probability was low but variable (Figure S5). This was expected due to the low recapture rates at both sites. There was a significant difference between the two sites (χ^2^=161, df=20, p<0.001). *Amblyomma americanum* individuals were more likely to be recaptured at CH1 over the study period, which may indicate a smaller population at the site (Figure S5b). With fewer ticks present, it is more likely that any one individual would be recaptured compared to a larger population.

***Minimum Number Alive***

A Minimum Number Alive assessment was performed with the capture histories for all mark-recapture data (Krebs 1966). Data were used from PT2 in 2013, 2016, and 2018; NN1 in 2014 and 2015; CH1 in 2014, 2015 and 2018, CH2 in 2015, HM1 in 2015, and PT3 in 2016. Ticks captured during a marking event were considered alive, as well as ticks captured in a previous marking event that were later recaptured. This likely underestimates the tick population size but allows for seasonal population variation to be taken into account. Because of differences in the frequency of observations between years, data were pooled for different time periods into the first and second half of each month, averaging tick density for the time period if multiple marking events occurred.

Minimum population densities were calculated from the transect length and minimum number alive assessments (Figure S6). A generalized least squares model was created with R package nlme (Pinheiro et al. 2013), with the square root of density per 10m^2^ as the response variable and site, life stage, year, species, and the interaction between site and life stage as the explanatory variables. Variance was modeled separately for every combination of site, life stage, species, and year to combat the heteroskedasticity of the data. A type III Wald Chi-square test (Wald 1943) was used to test the model, as the study had an unbalanced design. This tested if each response variable was significant to the model, after taking into account the effect of all other response variables (Table S2).

Site was not significantly correlated with the minimum population density (χ^2^=1.6876, df=5, p=0.89). A second generalized least squares model was created with human maintenance in place of site and analyzed in the same way (Table S3). Human maintenance of a site was also not correlated with the minimum population density (χ^2^=0.3931, df=1, p=0.53).

Density was most strongly correlated with year in both the site (χ^2^=121.4463, df=4, p>0.001) and human maintenance models (χ^2^=200.4082, df=4, p>0.001). The tick population density for all life stages and species combined was significantly higher in 2013 than all other years (Tukey HSD, p<0.001) (Figure S7). However, mark-recapture in 2013 was only done at PT2, so it is unknown if the high density was present at other locations that year. Minimum population density was also significantly different between species captured (χ^2^=43.4497, df=4, p>0.001) (Figure S6). *Amblyomma americanum* had a significantly higher population density than *D. variabilis* (Tukey HSD, p<0.001)*, I. affinis* (Tukey HSD, p<0.001), and *I. scapularis* (Tukey HSD, p=0.003), but not *A. maculatum* (Tukey HSD, p=0.07), which only had three ticks total across all samples.

**Discussion**

Field sites disturbed by humans were not found to have significantly different tick population densities than undisturbed sites (Table S3). This indicates that the maintenance of ecotones has little to no impact on tick populations when compared to other factors. Large-scale habitat fragmentation, like the preparation of land for agricultural use, may be necessary to see a difference in tick population density. Further, there was no significant difference between the population densities at the field sites sampled (Table S2). The survival and recapture probabilities were significantly different between PT2 and CH1 in 2018, but more years would be needed to determine if this difference is meaningful or not. Inter-annual variation was found to be the most significant factor in minimum population density of the factors tested. This variation is also seen in studies of *I. affinis* (Nadolny and Gaff 2018a) and *A. maculatum* (Nadolny and Gaff 2018b) in southeastern Virginia, and a study of *I. ricinus* in Central Russia (Korotkov et al. 2015). Further studies over longer time periods could be used to determine if density fluctuations are cyclic or are the result of an untested variable. It could not be determined whether all sites exhibited the same density fluctuations for each year, as no pair of sites was sampled together for two years.

The tick species captured in this study exhibited significant differences in population density (Table S2), survival probability (Figure S4), and recapture probability (Figure S5). *Amblyomma americanum* was the species with the highest density, which was significantly higher than *D. variabilis, I. affinis*, and *I. scapularis* densities. Understanding which species are the most prevalent in an area, as well as how much movement there is to and from a population, can help determine the risk of different tick-borne diseases in a certain area.

In this case study, human disturbance through ecotone maintenance was not found to significantly impact the tick populations of southeastern Virginia. This simple method allowed for multiple sites with and without human disturbance to be studied, and for the collected data to be analyzed with an open population and phenological variation in mind. Capture histories developed from multiple regular marking events can be used to test a variety of hypotheses, with CJS modeling allowing for the activities of the unmarked ticks to be estimated. Survival rate can be determined beyond measuring the longevity of marked ticks, and recapture probability can be predicted for each marking event, rather than an overall recapture rate. Minimum population assessments can also be created from the capture histories and can be adjusted for capture effort in the case of uneven time periods between marking events. While the underlying factors driving the inter-annual variation of the tick populations in this study may not be known, this method allowed for this important variation to be observed.

**References**

**Boyard, C., J. Barnouin, P. Gasqui, and G. Vourc’h**. **2007**. Local environmental factors characterizing *Ixodes ricinus* nymph abundance in grazed permanent pastures for cattle. Parasitology. 134: 987–994.

**Hurvich, C. M., and C.-L. Tsai**. **1989**. Regression and time series model selection in small samples. Biometrika. 76: 297–307.

**Korotkov, Y., T. Kozlova, and L. Kozlovskaya**. **2015**. Observations on changes in abundance of questing *Ixodes ricinus*, castor bean tick, over a 35-year period in the eastern part of its range (Russia, Tula region). Medical and Veterinary Entomology. 29: 129–136.

**Krebs, C. J.** **1966**. Demographic changes in fluctuating populations of *Microtus californicus*. Ecological Monographs. 36: 239–273.

**Laake, J. L.** **2013**. RMark: an R interface for analysis of capture-recapture data with MARK.

**Nadolny, R. M., and H. D. Gaff**. **2018a**. Natural history of *Ixodes affinis* in Virginia. Ticks and Tick-borne Diseases. 9: 109–119.

**Nadolny, R. M., and H. D. Gaff**. **2018b**. Natural history of *Amblyomma maculatum* in Virginia. Ticks and Tick-borne Diseases. 9: 188–195.

**Pinheiro, J., D. Bates, S. DebRoy, D. Sarkar, and R. C. Team**. **2013**. nlme: Linear and nonlinear mixed effects models. R package version. 3: 111.

**R Core Team**. **2019**. R: A Language and Environment for Statistical Computing. R Foundation for Statistical Computing, Vienna, Austria.

**Wald, A.** **1943**. Tests of statistical hypotheses concerning several parameters when the number of observations is large. Transactions of the American Mathematical Society. 54: 426–482.

**White, G.C., and K.P. Burnham. 1999.** Program MARK: survival estimation from populations of marked animals. Bird Study. 46: S120-S139.

**Figures and Tables**

**
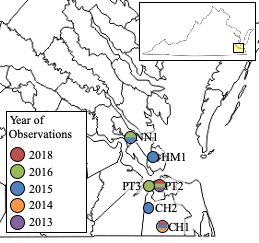
**

Figure S1: Location of all field sites where mark-recapture studies were conducted. Ticks were collected during the summer of each year indicated.

S2a.


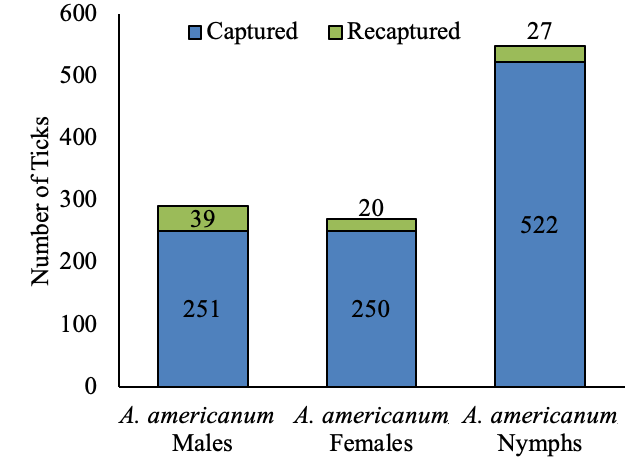


S2b.


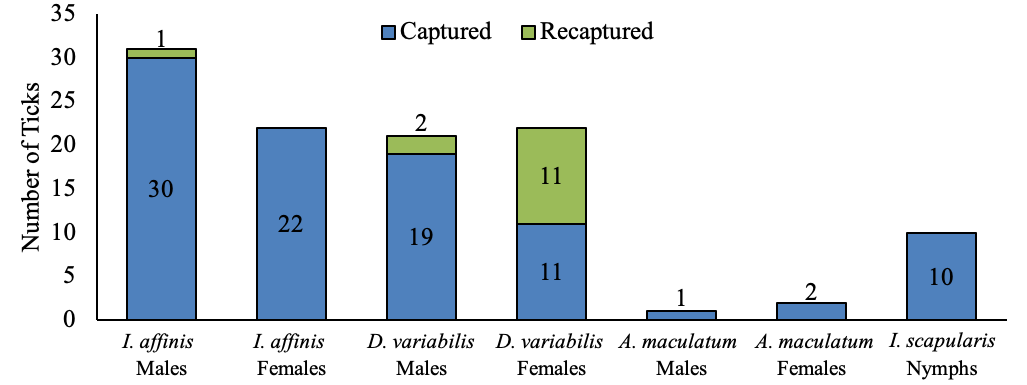


Figure S2: Number of ticks captured and recaptured by species and life stage. Note the difference in axes between *A. americanum*(S2a) and other tick species in the study (S2b).


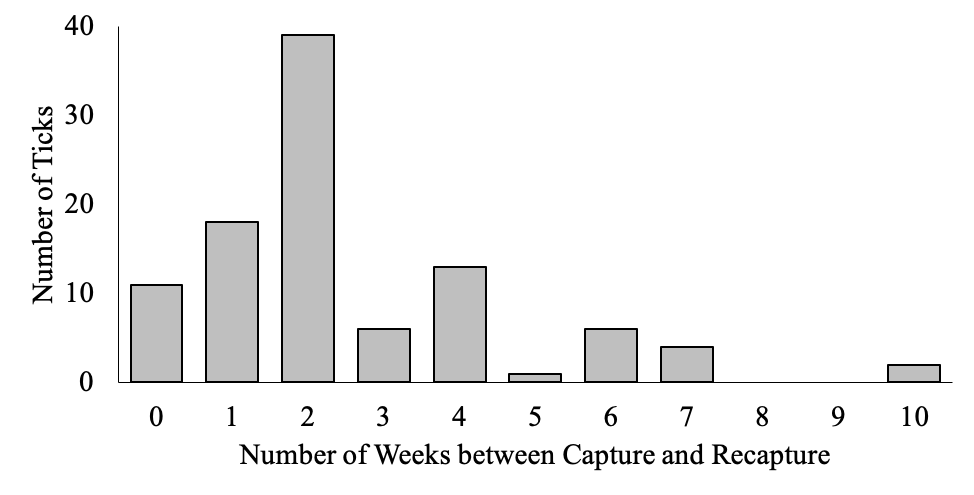


Figure S3: Longest time between the capture and recapture for each recaptured individual (n=100). Zero weeks refers to individuals only recaptured hours to three days after the initial marking event.

Table S1: Best CJS models for the 2018 mark-recapture data for sites PT2 (S1a) and CH1 (S1b). 65 other models were created for each site, but were not included as all had weights of 0. A 1 indicates a constant survival or recapture probability. Life stage, date, and species indicate variation in probability between each group within the variable.

S1a: PT2.

| Survival Probability | Recapture Probability | AIC_C_ | Weight | Deviance |
| --- | --- | --- | --- | --- |
| 1 | Life stage*date*species | -767.79598 | 0.8984499 | 1.184939 |
| Life stage*date*species | 1 | -763.43574 | 0.1015501 | 5.545177 |

S1b: CH1.

| Survival Probability | Recapture Probability | AIC_C_ | Weight | Deviance |
| --- | --- | --- | --- | --- |
| Species | Life stage*date | -575.75663 | 0.5657688 | 2.243372 |
| Life stage*date | Species | -575.22741 | 0.4342312 | 2.772589 |

S4a.

**PT2 2018**


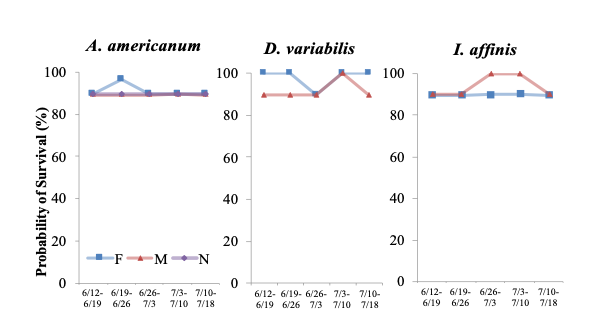


S4b.

**CH1 2018**


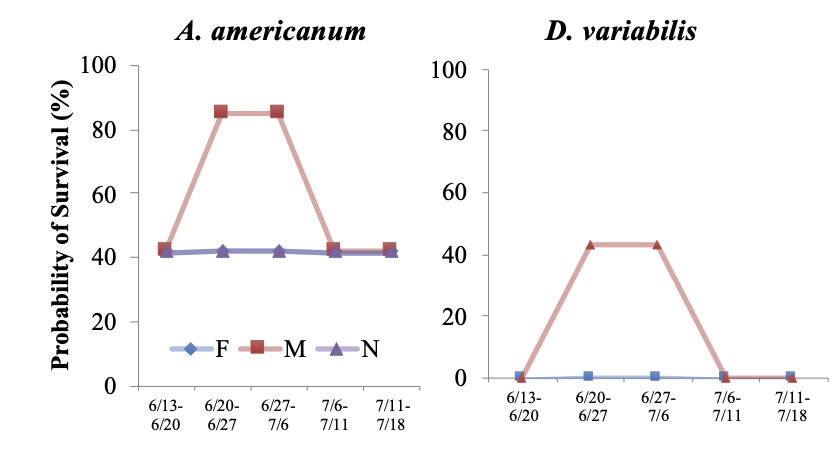


Figure S4: The probability of survival for ticks at PT2 and CH1 were estimated with CJS models for each site in 2018. Probability of survival represents the chance that a tick survives between two observation points and that a tick stays in the area studied. For all figures, F (blue), M (red), and N (purple) stand for adult female, adult male, and nymph, respectively.

S5a.


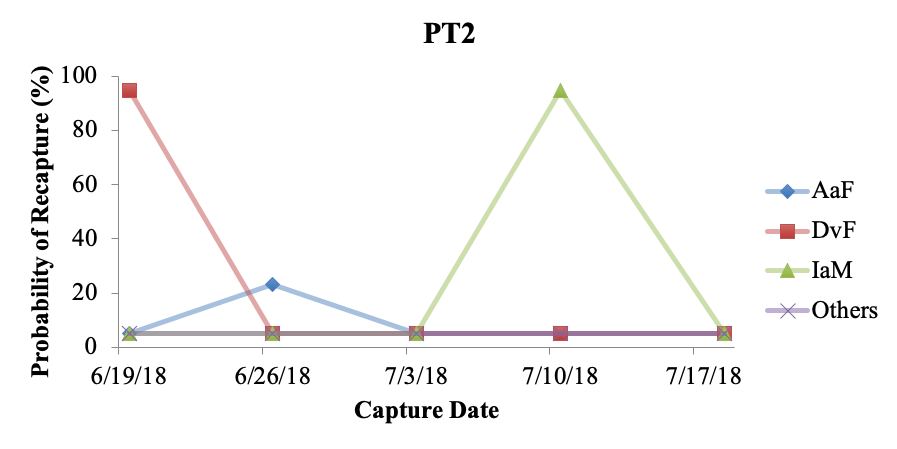


S5b.


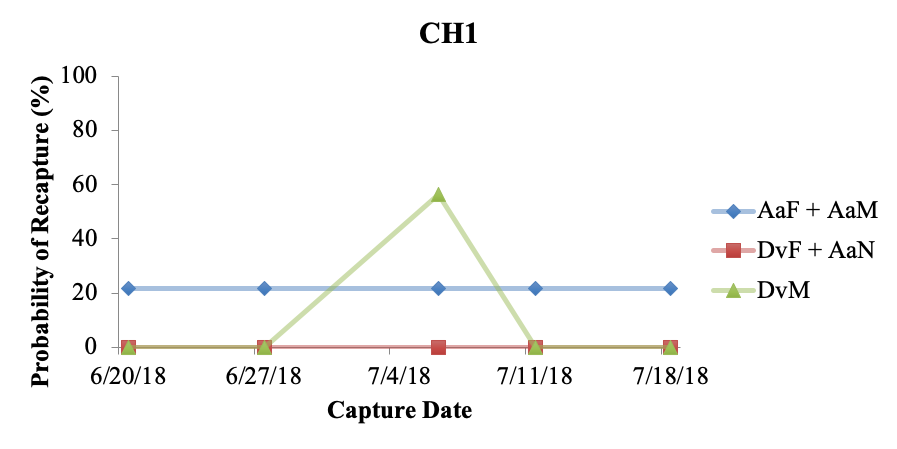


Figure S5: The probability of recapture for ticks at PT2 and CH1 were estimated with CJS models for each site in 2018. Abbreviations: *Ixodes affinis* (Ia), *Amblyomma americanum* (Aa), *Dermacentor variabilis* (Dv), males (M), females (F), and nymphs (N). Others in S5a includes *A. americanum* males and nymphs, *D. variabilis* males, and *I. affinis* females.


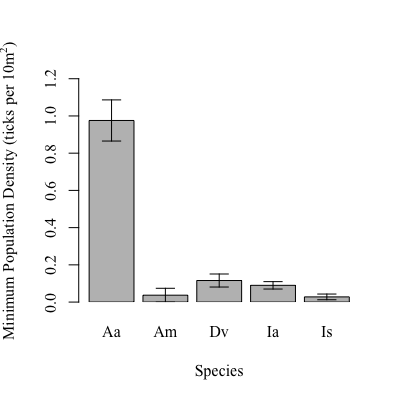


Figure S6: Minimum population density for all tick species. Abbreviations: *Amblyomma americanum* (Aa), *Amblyomma maculatum* (Am), *Dermacentor variabilis* (Dv), *Ixodes affinis* (Ia), *Ixodes scapularis* (Is). Data were pooled for all years from 2013-2018. Error bars indicate one standard deviation from the mean. *A. americanum* had a significantly higher population density than *D. variabilis, I. affinis*, and *I. scapularis* (Tukey HSD, p≤0.003), but not *A. maculatum* (Tukey HSD, p=0.07). n=560.

Table S2: Results of Analysis of Deviance of generalized least squares model with the square root of minimum population density per 10m^2^ as the response variable.

| Component | Degrees freedom | χ^2^ | P value |
| --- | --- | --- | --- |
| Intercept | 1 | 195.8847 | > 0.001 |
| Site | 5 | 1.6876 | 0.890 |
| Life Stage | 2 | 6.1529 | 0.046 |
| Year | 4 | 121.4463 | > 0.001 |
| Species | 4 | 43.4497 | > 0.001 |
| Site*Life Stage | 10 | 13.5221 | 0.196 |

n = 560.

Table S3: Results of Analysis of Deviance of generalized least squares model with the square root of minimum population density per 10m^2^ as the response variable.

| Component | Degrees freedom | χ^2^ | P value |
| --- | --- | --- | --- |
| Intercept | 1 | 207.8108 | > 0.001 |
| Human Maintenance | 1 | 0.3931 | 0.531 |
| Life Stage | 2 | 12.0359 | 0.002 |
| Year | 4 | 200.4082 | > 0.001 |
| Species | 4 | 66.8067 | > 0.001 |
| Human Maintenance*Life Stage | 2 | 0.7493 | 0.688 |

n = 560.


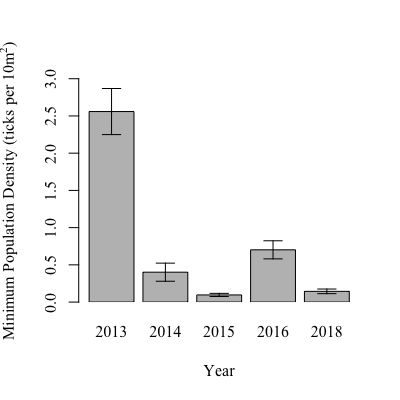


Figure S7: Minimum population density for each year. Ticks of all species and life stages are combined. Error bars indicate one standard deviation from the mean. 2013 was significantly different than all other years (Tukey HSD, p<0.001). n=560.
